# DNAAlignEditor: DNA alignment editor tool

**DOI:** 10.1186/1471-2105-9-154

**Published:** 2008-03-19

**Authors:** Hector Sanchez-Villeda, Steven Schroeder, Sherry Flint-Garcia, Katherine E Guill, Masanori Yamasaki, Michael D McMullen

**Affiliations:** 1 Division of Plant Sciences, University of Missouri, Columbia, MO 65211, USA; 2 USDA-ARS, Plant Genetics Research Unit, Columbia, MO 65211, USA; 3 Food Resources Education and Research Center, Kobe University, Hyogo, Japan; 4 USDA-ARS Bovine Functional Genomics Laboratory, Beltsville, MD 20705, USA

## Abstract

**Background:**

With advances in DNA re-sequencing methods and Next-Generation parallel sequencing approaches, there has been a large increase in genomic efforts to define and analyze the sequence variability present among individuals within a species. For very polymorphic species such as maize, this has lead to a need for intuitive, user-friendly software that aids the biologist, often with naïve programming capability, in tracking, editing, displaying, and exporting multiple individual sequence alignments. To fill this need we have developed a novel DNA alignment editor.

**Results:**

We have generated a nucleotide sequence alignment editor (DNAAlignEditor) that provides an intuitive, user-friendly interface for manual editing of multiple sequence alignments with functions for input, editing, and output of sequence alignments. The color-coding of nucleotide identity and the display of associated quality score aids in the manual alignment editing process. DNAAlignEditor works as a client/server tool having two main components: a relational database that collects the processed alignments and a user interface connected to database through universal data access connectivity drivers. DNAAlignEditor can be used either as a stand-alone application or as a network application with multiple users concurrently connected.

**Conclusion:**

We anticipate that this software will be of general interest to biologists and population genetics in editing DNA sequence alignments and analyzing natural sequence variation regardless of species, and will be particularly useful for manual alignment editing of sequences in species with high levels of polymorphism.

## Background

Maize is a highly polymorphic species with numerous single nucleotide polymorphisms (SNP) and insertion/deletions (InDel) commonly present among maize alleles [1,2]. One consequence of this level of polymorphism is that sequence alignments of maize genes constructed with standard software such as CLUSTAL W, Muscle, Dialign, MAFFT, T-Coffee, etc., must be visually inspected and manually edited to ensure that an optimal alignment is obtained. This problem, together with the need to make base calls and alignment decisions in regions of variable base quality, creates the need for a tool to assist the researcher in manual editing of sequence alignments. Researchers in communities other than maize also require user-friendly tools to facilitate input, processing, and output of the sequence diversity among individuals within the species. Although there are several improved methods [3] to generate alignments for multiple nucleotide sequences, there is a need to improve software for manual editing to evaluate and fix discrepancies. Our goal was to produce a sequence alignment tool (DNAAlignEditor) that provides an efficient path for editing multiple sequence alignments to improve alignment quality, tracking edited alignments, and exporting sequence files to external tools for analysis.

## Implementation

DNAAlignEditor is written in Visual Basic for the user interface, MS Access database for the stand-alone version or PostrgeSQL 8.0 database for the network version, and embedded ActiveX data object drivers for connectivity between the database and the user interface. DNAAlignEditor network version is designed to share alignment information when several users are simultaneously logged into the system and to manage alignments collected in the database. Regardless of whether the stand-alone or network version is used, the database aids in the tracking and manipulation of large numbers of sequences, and the relationship among genes, primers, amplicons, and population groups. The database is also required to track multiple versions of the same alignments and to combine alignments. Editing of a handful of gene alignments may be accomplished without the aid of a database; however, processing, editing, and finishing hundreds of alignments requires a more sophisticated system managed by a database.

**Figure 1:**
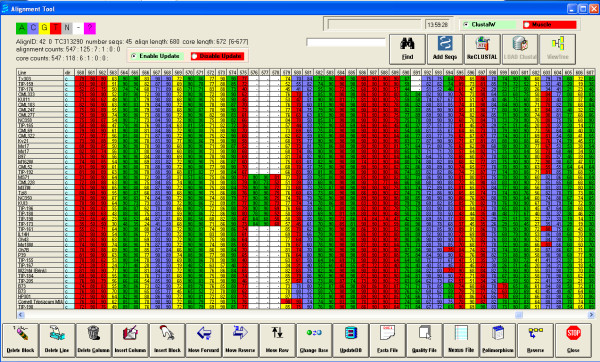
**DNAAlignEditor view of an example alignment.** The color coded cells represent the base calls and the numbers within the cells reflect the quality scores. Insertion/deletions are present in columns 576–578 and 591, and SNPs in columns 579, 580, 594, 597, 599 and 603. Below the alignment are the buttons used to manipulate sequence. To the left of the alignment are the names of the individuals displayed. Above the alignment are identification summary statistics on the alignment and additional buttons to manipulate sequence file and re-CLUSTAL functions.

DNAAlignEditor stores a set of interrelated tables in a relational database (DNADB) supporting alignment information to provide easy editing, querying, importing, and exporting function from genes, primers, populations, nucleotide sequence alignments, and quality scores as shown in the entity relationship diagram [see additional file 1]. The system is a user-friendly editing tool using grids, text boxes, combo boxes, and scroll lists to facilitate data access. DNAAlignEditor tool color codes nucleotide identity for ease in visual identification of sequence polymorphisms and displays the quality score associated with each base to aid in manual editing. The quality score-centric view is one of the major and unique features that distinguish DNAAlignEditor from other alignment programs (Figure 1).

DNAAlignEditor accepts alignments in three different formats and checks the consistency of the information before addition to the database [see additional file 2]. The first format uses the information from PHRAP ace files, and a file that defines the gene information associated with the alignment. The second format is a fasta format. The last format accepts a tab delimited text, file having a header with information related to gene, amplicon, and total bases followed by a nucleotide sequence row and a quality scores row (or no-quality where a default quality score of 15 is applied to all base calls) [see additional file 2].

The program validates the data before it is imported, i.e. that genes, amplicons, and populations exist in DNADB. For sequences without quality scores, the program assigns a default quality score of 15 for each base. This value can be changed in the configuration file. By definition, a *phred *quality score of Q ≥ 20 indicates that there is a less than 1 in 100 chance that the base call is incorrect. Consequently, "Q20" bases are considered "high quality" calls with corresponding low error probabilities [4,5]. By adding a standard quality score of Q = 15, we effectively flag those bases as unknown quality.

Once the alignment information is in the database, it can be edited in an intuitive, user-friendly interface. A user help file can be accessed by pressing the F1 key. The tool provides several features to edit information stored in the database such as moving individual sequences up and down for ease of fixing errors in multiple lines, moving bases from the 5' direction to the 3' direction and conversely, deleting and inserting individual bases and block of bases, and deleting and inserting entire columns in the alignment. DNAAlignEditor forms initial alignments by executing either of the integrated CLUSTAL W [6] or MUSCLE programs [7], and displays and prints phylogenies with TreeView [8]. The inclusion of a "Re-CLUSTAL" function allows editing to be preformed as a reiterative process, for instance rerunning CLUSTAL W after trimming end sequences or removing lower quality individual sequences for the initial alignment. DNAAlignEditor also allows the user to export fasta files with quality files and/or files compatible with DnaSP [9] for further analysis. Upon editing changes and saving, the tool will generate a new alignment while maintaining the original for further use or reference.

## Results and discussion

There are several software packages that produce nucleotide sequence alignments such as CLUSTAL W, Muscle, Dialign, MAFFT, and T-Coffee. However, all of these programs produce alignments that must be manually examined and edited for accuracy. By integrating a highly intuitive visual display with CLUSTAL functions, DNAAlignEditor allows the researcher move seamlessly between alignment and editing functions. The inclusion of quality scores allow the researcher to make informed decisions on editing InDel containing regions and accessing the certainty of unique nucleotide polymorphisms. The database functions promote efficient tracking of large sets of primers, genes, amplicons; and the relationships among these components of diversity sequencing projects. It is the focus on intuitive manual editing functions, inclusion of quality scores, and power of tracking components in large project that distinguish the software from other available for alignment functions. While a variety of alignment editors are now available include BioEdit [10], JalView [11], and INTERALIGN [12]. Of these only JalView, like DNAAlignEditor, is open source. None of the other programs is as intuitive to the naïve user nor uses the quality score feature central to DNAAlignEditor. In addition, the database functions of DNAAlignEditor for tracking the components of a re-sequencing projects makes DNAAlignEditor more appropriate for large DNA sequence diversity projects.

This software has been successfully used by the National Science Foundation funded "Molecular and Functional Diversity of the Maize Genome" group at the University of Missouri [13], University of Wisconsin, and Cold Spring Harbor Laboratories for manual editing of alignments. DNAAlignEditor was essential for tracking and exporting over 4,000 alignments are currently stored in DNADB with more than 50,000 sequences from several maize, teosinte, and Tripsacum accessions. In addition the amplicon merge feature was very helpful for extended sequencing of genes of particular interest to the group. The finished alignments are displayed in a comprehensive graphical user interface tool included in panzea site [14].

## Conclusion

We have developed a nucleotide sequence alignment editor (DNAAlignEditor) that provides an intuitive, user-friendly tool for manual editing of multiple sequence alignments which can be used for different species to allow researchers to visualize and edit the alignment information in a straight forward way, and realign the sequences using the CLUSTAL W or Muscle software, as well as export information to different packages for further analysis.

## Availability and requirements

**Project name**: DNAAlignEditor executable and source code are freely available at  [See additional files 3, 4]

**Contact**: sanchezvilledah@missouri.edu

**Operating systems**: Windows 95/98/NT/2000/XP; can run on Intel Mac using Parallels

**Programming language**: Visual Basic 6.0

## Abbreviations

CLUSTAL W: general purpose multiple sequence alignment program for DNA or protein sequences. Muscle: multiple sequence alignment comparison by log-expectation program.

MAFFT: multiple sequence alignment program. T-COFFE: multiple sequence alignment program.

ActiveX: is the name Microsoft has given to a set of "strategic" object-oriented programming technologies and tools. SNP: single nucleotide polymorphism. InDel: insertion-deletion polymorphism. MS Access: Microsoft Access. PHRAP: is a program for assembling shotgun DNA sequence data. Phred: is a program that reads DNA sequencing trace files, calls bases, and assigns a quality value to each called base.

## Authors' contributions

HS-V elaborated the design, performed programming, wrote the documentation for the SNPAlign Editor tool, and prepared the manuscript. SS assisted in programming and design. SF-G, KEG and MY contributed design ideas and testing. MDM contributed to design and supervised the project. All authors have read and approved the manuscript.

## Supplementary Material

Additional file 1 Entity-relationship diagram for DNADB database. The first column of each table contains the field name and the second column contains the data type. The underlined row indicates that the field is a primary key. Click here for file

Additional file 2 DNA alignment editor tool file formats. Definition of file formats accepted to import DNA alignments to DNAAlignEditor tool. Click here for file

Additional file 3 DNA alignment editor tool source core. Source code for DNA align editor tool in zip format. Click here for file

Additional file 4 DNA alignment tool installation instructions. Installation steps for DNAAlignEditor tool. Click here for file
