# Supplementary figures and images for: DNAAlignEditor: DNA alignment editor tool
Source: BMC Bioinformatics. 2008 Mar 19;9:154. doi: 10.1186/1471-2105-9-154 (PMC2322986; doi:10.1186/1471-2105-9-154)

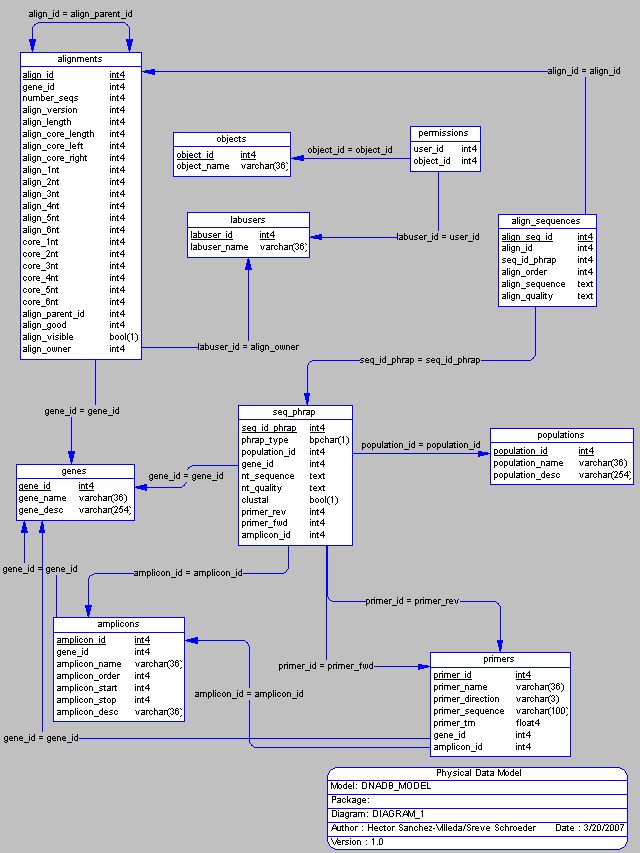

Supplement: Additional file 1 — Entity-relationship diagram for DNADB database. The first column of each table contains the field name and the second column contains the data type. The underlined row indicates that the field is a primary key. [file 1471-2105-9-154-S1.jpeg]
