# Supplementary material for: DNAAlignEditor: DNA alignment editor tool
Source: BMC Bioinformatics. 2008 Mar 19;9:154. doi: 10.1186/1471-2105-9-154 (PMC2322986; doi:10.1186/1471-2105-9-154)
Supplement: Additional file 2 — DNA alignment editor tool file formats. Definition of file formats accepted to import DNA alignments to DNAAlignEditor tool. [file 1471-2105-9-154-S2.doc]

File2. File formats and process to import alignments to DNAAlignEditor tool.

IMPORT FORMAT 1

Steps to load ACE files using the DNAAlignEditor tool.

1) Click on Data and Import Alignments to get Import Alignments form

2) Click either on Phrap or One directory radio button

3) Select on the drop down box of your directory the location of the ace files. (All the ace files for the alignment

must be located in the same directory). A segment of the ace file format is as follows:

AS 1 2

CO Contig1 672 2 95 U

tcttcTTATGTTCCCAGAGTTGGGTATTACAACATTCATGTATGCATTCT

AGGATTTCCATTAGTATCAATCTTGTAGTATTTGTTCCTTCATTGTCAAA

TCACTTCTCATCTAACTACTATGCTTGTTTAACCAGCAGAACAATACTAC

AACAATATCCATTTATAAAGGCTTTGATAGCAAACTTTACATATTCATAT

CATGTTAAGGTTGTCACATGTGTAAAGGTGAAGAGATCATGCATGCCATT

CCACATAAATGAAAAGAATTCCTATATAAAAATGACATGTTTTGTTGTAG

GTAGTGGAAATTATCTTTCTAGCAAAGACCATATAATCCGATAAAGCTGA

TAACTAAATGTCAAAATCGAGTAGGTGCCATATCATCTATATCTTATCTG

TTGTTTGGAAAAATACAAAATCCAAAAAAATATATGAGATCTCACCTATA

TAAATAGCTCCCAAATCAGTAGTTAATACATCTCCCATAATATTTTCAGA

ATTTAGAAACACACCAAGCGAAGCGCACTAGCAACGACCTAACAACAATG

GCTACCAAGATATTAGCCCTCCTTGCGCTTCTTTCCCTTTTAGTGAACGC

AACAAATGCGTTCATTATTCCACAGTGCTCACTTGCTCCTAGTGCCAGTA

TTCCACAgttcctcacacaaaa

BQ

0 0 0 0 0 59 59 59 59 57 48 48 48 48 47 47 43 41 37 37 37 37 42 59 43 43 43 43 43 43 43 43 43 58 58 58 72 87 63 68 76 76 69 78 74 83 83 90 90 90

90 90 81 87 87 90 83 84 84 90 74 81 75 74 65 77 74 84 82 70 70 63 74 67 59 67 74 76 84 90 90 90 90 90 90 90 90 90 90 90 90 90 90 90 87 87 87 90 90 90

90 90 90 90 90 90 90 90 90 90 77 77 73 90 89 89 90 90 90 87 90 90 90 90 90 90 90 90 90 90 90 90 90 90 90 90 90 90 90 83 88 88 90 90 86 76 83 84 90 90

90 90 90 90 90 90 90 90 90 90 90 90 90 90 90 90 90 90 90 90 90 90 90 90 90 90 90 90 90 90 90 90 90 90 90 90 90 90 90 90 90 90 90 90 90 90 90 90 90 90

90 90 90 90 90 90 90 90 90 90 90 90 90 90 90 90 90 82 82 70 76 76 90 86 90 86 86 70 70 67 79 77 77 77 81 81 90 87 90 83 83 81 88 74 83 90 90 90 90 90

83 90 90 90 90 90 90 90 90 90 90 90 90 90 90 90 90 90 87 90 90 83 83 90 90 90 90 90 90 90 90 90 90 88 86 82 78 78 83 90 90 90 90 90 90 90 90 90 90 90

90 90 90 90 81 81 90 90 90 90 90 90 90 90 90 90 90 90 90 90 79 79 90 90 90 90 90 90 90 90 90 90 90 90 90 90 90 90 90 90 90 90 90 90 86 78 74 74 86 87

86 90 90 90 83 87 75 76 76 88 90 90 90 90 90 90 87 78 84 82 86 90 90 78 78 82 82 90 90 90 90 90 90 90 90 73 89 90 90 90 90 90 87 87 90 90 90 90 82 90

72 81 90 90 90 90 90 90 90 90 90 90 90 90 90 90 90 90 90 90 90 90 90 90 90 90 90 90 90 90 90 90 90 90 90 90 90 90 90 90 90 90 90 90 87 90 90 90 90 90

90 90 90 90 90 90 90 90 90 90 90 90 90 90 90 90 90 90 90 90 90 90 90 90 90 90 90 90 90 90 90 90 90 90 90 90 90 90 90 90 90 90 90 90 90 90 90 90 90 90

89 90 87 90 87 85 85 90 90 90 90 90 90 90 90 90 90 90 90 90 90 83 90 76 84 84 90 83 89 79 80 80 90 90 90 90 90 90 90 90 90 90 84 84 70 75 82 84 90 90

90 90 90 90 90 90 90 90 90 90 90 90 90 90 90 90 90 90 84 84 85 85 90 90 90 90 90 90 90 90 90 90 90 90 90 90 90 90 90 57 57 41 42 42 43 59 62 63 63 83

74 74 60 58 58 57 59 57 48 39 39 39 37 43 43 47 48 57 48 47 39 35 30 30 34 41 43 41 40 43 43 43 47 48 48 48 43 43 28 28 23 37 37 39 39 39 39 39 39 47

47 43 33 28 28 24 20 16 0 0 0 0 0 0 0 0 0 0 0 0 0 0

...

4) Click on Select Populations File button (The populations file format contains the # character in the first row)

each of the next rows contains the population name, a tab, and the name of the ace file as follows:

#populations

P39 al_t1.ace.1

B73 al_t2.ace.1

EH1 al_t3.ace.1

EH10 al_t4.ace.1

EH11 al_t5.ace.1

Note. Once you select the populations file, it will be validated against the population table; the populations

P39, B73, EH1, EH10 and EH11 must exist in populations table.

5) Click on Find Gene Name button to select the gene, a grid with the genes in the database will appear,

double click on it to choose the gene.

6) Click on Find Amplicon Name button to select the Amplicon, a grid with the amplicons that belong to

the selected gene will appear, double click on it to choose the amplicon.

7) Click on Validate button to validate gene name and amplicon name.

8) Click on Select error log file button and give a file name, to write errors generated in the import process.

9) Finally click on OK button to get the list of popluations, directory and files in a new form ready to

update the database. Verify the information displayed and click on Select button to load the alignment into the database.

10) Click on Data and edit alignment tool to check your alignment in the database and double click on

the alignment to open it for editing.

IMPORT FORMAT 2

Steps to load Fasta format files using the DNAAlignEditor tool.

1) Click on Data and Import Alignments to get the Import Alignments form

2) Click on Fasta file radio button

3) Click on Find Gene Name button to select the gene, a grid with the genes in the database will appear,

double click on it to choose the gene.

4) Click on Find Amplicon Name button to select the amplicon, a grid with the amplicons that belong to

the selected gene will appear, double click on it to choose the amplicon.

5) Click on Validate button to validate gene name and amplicon name.

6) Click on Select Input File. The fasta file format as follows:

>EH1

ATCAATTGTTCAATAAATTACTAAAGATCGTTACTACTGTTGATGCTCTTAACAGGACCA

TCTCTTGATAGATGAGATTCACGATTTGAATCGAAAGGTCTGTAATATGGTGAATTTATA

GTTCTGTGTTATGTGTTTTATTTGATTTAGTAGCACTGACTAACCACTTTGTTGGTCACA

GGCAAGTTTATTTCACCAAGAAAATACAGACTTGTACAATAAGATCAACCTGATTCGCCA

AGAAAATGATGAGTTACATAAAAAGGTAGCTGATGAATTGAATAAAAATACCACTAAGTA

ACTGCCTAACCACACATATGACATGATATTAATTGTTCTCAGATCTATGAGACTGAAGGA

CCAAGTGGAGTTAATCGGGAGTCACCGACTCCATTCAACTTTGCAGTAGTAGAAACCAGA

GATGTTCCTGTGCAACTTGAACTCAGCACACTGCCACAGCAAAATAACATTGAGCCATCT

ACTGCTCCTAAGCTAGGGTATAACATTTATTCTCT--------CTATATATTTCAATTGG

TTAACTAGTGTTGAC--CAGTGCTATAATGAGTTGCTAAACACATGCAGATTGCAATTAA

TTCCATGAAGAAGAGTAAAACTGCCGTCTTATGATGCTGAAGGAAACTATTTATTGTGAA

GAGATGATACTCAGAGAAAGACATATTTGTGGCAGGGAGATTTGA

>EH10

ATCAATTGTTCAATAAATTACTAAAGATCGTTACTACTGTTGATGCTCTTAACAGGACCA

TCTCTTGATAGATGAGATTCATGATTTGAATCGAAAGGTCTGTAATATGGTGAATTTATA

GTTCTCTGTTATGTGTTTTATTTGATTTAGTAGCACTGACTAACCACTTTGTTGGTCACA

GGCAAGTTTATTTCACCAAGAAAATACAGACTTGTACAATAAGATCAACCTGATTCGCCA

AGAAAATGATGAGTTACATAAAAAGGTAGCTGATGAATTGAACAAAAATACCACTAAGTA

ACTGCCTAACCACACATATGACATGATATTAATTGTTCTCAGATCTATGAGACTGAAGGA

CCAAGTGGAGTTAATCGGGAGTCACCGACTCCATTCAACTTTGCAGTAGTAGAAACCAGA

GATGTTCCTGTGCAACTTGAACTCAGCACACTGCCACAGCAAAATAATATTGAGCCATCT

ACTGCTCCTAAGCTAGGGTACAACATTTATTCTCT--------CTATATATTTCAATTGG

TTAACTAGTGTTGAC--CAGTGCTATAATGAGTTGCTAAACACATGCAGATTGCAATTAA

TTCCATGAAGAAGAGTAAAACTGCCGTCTTATGATGCTGAAGGAAACTATTTATTGTGAA

GAGATGATACTCAGAGAAAGACATATTTGTGGCAGGGAGATTTGA

>EH11

ATCAATTGTTCAATAAATTACTAAAGATCGTTACTACTGTTGATGCTCTTAACAGGACCA

TCTCTTGATAGATGAGATTCACGATTTGAATCGAAAGGTCTGTAATATGGTGAATTTATA

GTTCTGTGTTATGTGTTTTATTTGATTTAGTAGCACTGACTAACCACTTTGTTGGTCACA

GGCAAGTTTATTTCACCAGGAAAATACAGACTTGTACAATAAGATCAACCTGATTCGCCA

AGAAAATGATGAGTTACATAAAAAGGTAGCTGATGAATTGAACAAAAATACCACTAAGTA

ACTGTCTAACCACACATATGACATGATATTAATTGTTCTCAGATCTATGAGACTGAAGGA

CCAAGTGGAGTTAATCGGGAGTCACCAACTCCATTCAACTTTGCAGTAGTAGAAACCAGA

GATGTTCCTGTGCAACTTGAACTCAGCACACTGCCACAACAAAATAACATTGAGCCATCT

ACTGCTCCTAAGCTAGGGTATAACATTTATTCTCT--------CTATATATTTCAATTGG

TTAACTAGTGTTGAC--CAGTGCTATAATGAGTTGCTAAACACATGCAGATTGCAATTAA

TTCCATGAAGAAGAGTAAAACTGCCGTCTTATGATGCTGAAGGAAACTATTTATTGTGAA

GAGATGATACTCAGAGCAAGACATATTTGTGGCAGGGAGATTTGA

7) Click on Select error log file button and give a file name, to write errors generated in the import process.

8) Finally click on OK button to update the database.

IMPORT FORMAT 3.

Steps to load standard format files using the DNAAlignEditor tool.

1) Click on Data and Import Alignments to get Import Alignments form

2) Click on Standard or One file radio button

4) Click on Select Input File. The standard input file format contains the # character in the first row

followed by a gene name, a tab, and an amplicon name; the second row contain the > character followed by

population name, a tab, the total number of bases, a tab, c/n (c to cluster alignments uses ClustalW program as default; n uses Muscle program as default), a tab, and yes/no to indicate if the gene is already aligned

the third row contains the nucleotide sequence, and the fourth row contains either the quality scores, or no-qualityscores, and

in the last row the word "End" to indicate the end of sequences to import. You can have multiple genes/amplicons

by using the # character to separate them as follows:

#MAGI_32955 MAGI_32955.1

>L317 nss 473 c yes

ANTTGCTACNNTGGATGAGCACNACGTCCAANANCCANGGNNCAGGGNANGGNNGGNCAGGTAANCGNAGGGANTTCCNCNGGCNACAAGGNAGGAACNNGCCGGGGGGGTNGANGGNGAGGNNGGCCTACCNNGGCNCCCNCNCTCTCTCTNTTTCAGCNCTNCCTANCNACTCNNTNTTCTNGGGGCTCGACCCNGGTGTGNTTCGNGGCGAGNCCNCANNATGTGNNATTTTANCCCCGAAATCNGGTGACNACANCCCNCCNAAANCCNNGGGAAANACNCCNANCNGGNNTCTTNNTATTTTCTNTTGGCNCNCCCCNNTTCTNTNGGNGAGNTNNANNATNCCANACNGGNGGGNCCCTCNNCTAGAGANAGCAATANCNNCACGCAAAGCNTACGGNCNGANNGGNTACCCGNTTGGNCNGAGTGGTNGAGGAAANTCGTGNNNTAAAANCAGGGTTTANGGCCNT

15 10 7 16 9 19 20 1 17 18 20 19 1 1 21 2 14 13 17 18 1 22 22 17 18 1 22 22 17 20 29 18 21 1 19 2 26 14 26 14 26 17 21 22 22 22 2 18 1 22 15 10 7 16 9 19 20 1 17 18 20 19 1 1 21 2 14 13 17 18 1 22 22 17 18 1 22 22 17 20 29 18 21 1 19 2 26 14 26 14 26 17 21 22 22 22 2 18 1 22 15 10 7 16 9 19 20 1 17 18 20 19 1 1 21 2 14 13 17 18 1 22 22 17 18 1 22 22 17 20 29 18 21 1 19 2 26 14 26 14 26 17 21 22 22 22 2 18 1 22 15 10 7 16 9 19 20 1 17 18 20 19 1 1 21 2 14 13 17 18 1 22 22 17 18 1 22 22 17 20 29 18 21 1 19 2 26 14 26 14 26 17 21 22 22 22 2 18 1 22 15 10 7 16 9 19 20 1 17 18 20 19 1 1 21 2 14 13 17 18 1 22 22 17 18 1 22 22 17 20 29 18 21 1 19 2 26 14 26 14 26 17 21 22 22 22 2 18 1 22 15 10 7 16 9 19 20 1 17 18 20 19 1 1 21 2 14 13 17 18 1 22 22 17 18 1 22 22 17 20 29 18 21 1 19 2 26 14 26 14 26 17 21 22 22 22 2 18 1 22 15 10 7 16 9 19 20 1 17 18 20 19 1 1 21 2 14 13 17 18 1 22 22 17 18 1 22 22 17 20 29 18 21 1 19 2 26 14 26 14 26 17 21 22 22 22 2 18 1 22 15 10 7 16 9 19 20 1 17 18 20 19 1 1 21 2 14 13 17 18 1 22 22 17 18 1 22 22 17 20 29 18 21 1 19 2 26 14 26 14 26 17 21 22 22 22 2 18 1 22 15 10 7 16 9 19 20 1 17 18 20 19 1 1 21 2 14 13 17 18 1 22 22 17 18 1 22 22 17 20 29 18 21 1 19 2 26 14 26 14 26 17 21 22 22 22 2 18 1 22 15 10 7 16 9 19 20 1 17 18 20 19 1 1 21 2 14 13 17 18 1 22 22

>P39 900 f yes

NNTTGCTANNNTGGATNAGCACNACGTCCAANANCCANGGNNCAGGGNANGGNNGNNCAGGTAANCGNAGGNANTTCCNCNGGCNACNAGGNAGNANCNNGCCGGGGGGGTNNANGGNGAGGNNGNCCNACCNNGGCNCCCNCNCTCTCTCTNTTTCAGCNCTNCNTANNNNCTCTNTNTTCTNGGGGCTCNACCCNGGTGNGNTTCGNGGCGAGNNCNCANNATGTGNNANTTTANCCCCGNAATCNGGTGANAACANCCCNCCNAAANCCNAGGGNAANACNCCNANCNGGCNTNTTNNTATTTTCTNTNGGCNCNCCCCNNTTCTNTNGGNGAGNTNGANNATNCCANACNGGNGGGNCCCTCNNCTAGAGANAGCAATANCNNCACNCAAAGCNTACCGNCNGACNGGNTACCNGNTTGGNCCGAGNGGNNGAGGNAANTCGTGNNNNTTGCTANNNTGGATNAGCACNACGTCCAANANCCANGGNNCAGGGNANGGNNGNNCAGGTAANCGNAGGNANTTCCNCNGGCNACNAGGNAGNANCNNGCCGGGGGGGTNNANGGNGAGGNNGNCCNACCNNGGCNCCCNCNCTCTCTCTNTTTCAGCNCTNCNTANNNNCTCTNTNTTCTNGGGGCTCNACCCNGGTGNGNTTCGNGGCGAGNNCNCANNATGTGNNANTTTANCCCCGNAATCNGGTGANAACANCCCNCCNAAANCCNAGGGNAANACNCCNANCNGGCNTNTTNNTATTTTCTNTNGGCNCNCCCCNNTTCTNTNGGNGAGNTNGANNATNCCANACNGGNGGGNCCCTCNNCTAGAGANAGCAATANCNNCACNCAAAGCNTACCGNCNGACNGGNTACCNGNTTGGNCCGAGNGGNNGAGGNAANTCGTGNN

no-qualityscores

#MAGI_2833 MAGI_2833.1

>EH10 900 f yes

NNTTGCTANNNTGGATNAGCACNACGTCCAANANCCANGGNNCAGGGNANGGNNGNNCAGGTAANCGNAGGNANTTCCNCNGGCNACNAGGNAGNANCNNGCCGGGGGGGTNNANGGNGAGGNNGNCCNACCNNGGCNCCCNCNCTCTCTCTNTTTCAGCNCTNCNTANNNNCTCTNTNTTCTNGGGGCTCNACCCNGGTGNGNTTCGNGGCGAGNNCNCANNATGTGNNANTTTANCCCCGNAATCNGGTGANAACANCCCNCCNAAANCCNAGGGNAANACNCCNANCNGGCNTNTTNNTATTTTCTNTNGGCNCNCCCCNNTTCTNTNGGNGAGNTNGANNATNCCANACNGGNGGGNCCCTCNNCTAGAGANAGCAATANCNNCACNCAAAGCNTACCGNCNGACNGGNTACCNGNTTGGNCCGAGNGGNNGAGGNAANTCGTGNNNNTTGCTANNNTGGATNAGCACNACGTCCAANANCCANGGNNCAGGGNANGGNNGNNCAGGTAANCGNAGGNANTTCCNCNGGCNACNAGGNAGNANCNNGCCGGGGGGGTNNANGGNGAGGNNGNCCNACCNNGGCNCCCNCNCTCTCTCTNTTTCAGCNCTNCNTANNNNCTCTNTNTTCTNGGGGCTCNACCCNGGTGNGNTTCGNGGCGAGNNCNCANNATGTGNNANTTTANCCCCGNAATCNGGTGANAACANCCCNCCNAAANCCNAGGGNAANACNCCNANCNGGCNTNTTNNTATTTTCTNTNGGCNCNCCCCNNTTCTNTNGGNGAGNTNGANNATNCCANACNGGNGGGNCCCTCNNCTAGAGANAGCAATANCNNCACNCAAAGCNTACCGNCNGACNGGNTACCNGNTTGGNCCGAGNGGNNGAGGNAANTCGTGNN

no-qualityscores

>EH16 473 c yes

ANTTGCTACNNTGGATGAGCACNACGTCCAANANCCANGGNNCAGGGNANGGNNGGNCAGGTAANCGNAGGGANTTCCNCNGGCNACAAGGNAGGAACNNGCCGGGGGGGTNGANGGNGAGGNNGGCCTACCNNGGCNCCCNCNCTCTCTCTNTTTCAGCNCTNCCTANCNACTCNNTNTTCTNGGGGCTCGACCCNGGTGTGNTTCGNGGCGAGNCCNCANNATGTGNNATTTTANCCCCGAAATCNGGTGACNACANCCCNCCNAAANCCNNGGGAAANACNCCNANCNGGNNTCTTNNTATTTTCTNTTGGCNCNCCCCNNTTCTNTNGGNGAGNTNNANNATNCCANACNGGNGGGNCCCTCNNCTAGAGANAGCAATANCNNCACGCAAAGCNTACGGNCNGANNGGNTACCCGNTTGGNCNGAGTGGTNGAGGAAANTCGTGNNNTAAAANCAGGGTTTANGGCCNT

no-qualityscores

#MAGI_97488 MAGI_97488.1

>EH14 473 c yes

ANTTGCTACNNTGGATGAGCACNACGTCCAANANCCANGGNNCAGGGNANGGNNGGNCAGGTAANCGNAGGGANTTCCNCNGGCNACAAGGNAGGAACNNGCCGGGGGGGTNGANGGNGAGGNNGGCCTACCNNGGCNCCCNCNCTCTCTCTNTTTCAGCNCTNCCTANCNACTCNNTNTTCTNGGGGCTCGACCCNGGTGTGNTTCGNGGCGAGNCCNCANNATGTGNNATTTTANCCCCGAAATCNGGTGACNACANCCCNCCNAAANCCNNGGGAAANACNCCNANCNGGNNTCTTNNTATTTTCTNTTGGCNCNCCCCNNTTCTNTNGGNGAGNTNNANNATNCCANACNGGNGGGNCCCTCNNCTAGAGANAGCAATANCNNCACGCAAAGCNTACGGNCNGANNGGNTACCCGNTTGGNCNGAGTGGTNGAGGAAANTCGTGNNNTAAAANCAGGGTTTANGGCCNT

15 10 7 16 9 19 20 1 17 18 20 19 1 1 21 2 14 13 17 18 1 22 22 17 18 1 22 22 17 20 29 18 21 1 19 2 26 14 26 14 26 17 21 22 22 22 2 18 1 22 15 10 7 16 9 19 20 1 17 18 20 19 1 1 21 2 14 13 17 18 1 22 22 17 18 1 22 22 17 20 29 18 21 1 19 2 26 14 26 14 26 17 21 22 22 22 2 18 1 22 15 10 7 16 9 19 20 1 17 18 20 19 1 1 21 2 14 13 17 18 1 22 22 17 18 1 22 22 17 20 29 18 21 1 19 2 26 14 26 14 26 17 21 22 22 22 2 18 1 22 15 10 7 16 9 19 20 1 17 18 20 19 1 1 21 2 14 13 17 18 1 22 22 17 18 1 22 22 17 20 29 18 21 1 19 2 26 14 26 14 26 17 21 22 22 22 2 18 1 22 15 10 7 16 9 19 20 1 17 18 20 19 1 1 21 2 14 13 17 18 1 22 22 17 18 1 22 22 17 20 29 18 21 1 19 2 26 14 26 14 26 17 21 22 22 22 2 18 1 22 15 10 7 16 9 19 20 1 17 18 20 19 1 1 21 2 14 13 17 18 1 22 22 17 18 1 22 22 17 20 29 18 21 1 19 2 26 14 26 14 26 17 21 22 22 22 2 18 1 22 15 10 7 16 9 19 20 1 17 18 20 19 1 1 21 2 14 13 17 18 1 22 22 17 18 1 22 22 17 20 29 18 21 1 19 2 26 14 26 14 26 17 21 22 22 22 2 18 1 22 15 10 7 16 9 19 20 1 17 18 20 19 1 1 21 2 14 13 17 18 1 22 22 17 18 1 22 22 17 20 29 18 21 1 19 2 26 14 26 14 26 17 21 22 22 22 2 18 1 22 15 10 7 16 9 19 20 1 17 18 20 19 1 1 21 2 14 13 17 18 1 22 22 17 18 1 22 22 17 20 29 18 21 1 19 2 26 14 26 14 26 17 21 22 22 22 2 18 1 22 15 10 7 16 9 19 20 1 17 18 20 19 1 1 21 2 14 13 17 18 1 22 22

>EH15 900 f yes

NNTTGCTANNNTGGATNAGCACNACGTCCAANANCCANGGNNCAGGGNANGGNNGNNCAGGTAANCGNAGGNANTTCCNCNGGCNACNAGGNAGNANCNNGCCGGGGGGGTNNANGGNGAGGNNGNCCNACCNNGGCNCCCNCNCTCTCTCTNTTTCAGCNCTNCNTANNNNCTCTNTNTTCTNGGGGCTCNACCCNGGTGNGNTTCGNGGCGAGNNCNCANNATGTGNNANTTTANCCCCGNAATCNGGTGANAACANCCCNCCNAAANCCNAGGGNAANACNCCNANCNGGCNTNTTNNTATTTTCTNTNGGCNCNCCCCNNTTCTNTNGGNGAGNTNGANNATNCCANACNGGNGGGNCCCTCNNCTAGAGANAGCAATANCNNCACNCAAAGCNTACCGNCNGACNGGNTACCNGNTTGGNCCGAGNGGNNGAGGNAANTCGTGNNNNTTGCTANNNTGGATNAGCACNACGTCCAANANCCANGGNNCAGGGNANGGNNGNNCAGGTAANCGNAGGNANTTCCNCNGGCNACNAGGNAGNANCNNGCCGGGGGGGTNNANGGNGAGGNNGNCCNACCNNGGCNCCCNCNCTCTCTCTNTTTCAGCNCTNCNTANNNNCTCTNTNTTCTNGGGGCTCNACCCNGGTGNGNTTCGNGGCGAGNNCNCANNATGTGNNANTTTANCCCCGNAATCNGGTGANAACANCCCNCCNAAANCCNAGGGNAANACNCCNANCNGGCNTNTTNNTATTTTCTNTNGGCNCNCCCCNNTTCTNTNGGNGAGNTNGANNATNCCANACNGGNGGGNCCCTCNNCTAGAGANAGCAATANCNNCACNCAAAGCNTACCGNCNGACNGGNTACCNGNTTGGNCCGAGNGGNNGAGGNAANTCGTGNN

no-qualityscores

End

5) Click on Select error log file button and give a file name, to write errors generated in the import process.

6) Finally click on OK button to update the database.

Note. The Standard format file can be generated either in an editor tool, or using the DNAAlignEditor tool network version which exports alignments

already loaded to the stand-alone version.

For comments email: Hector Sanchez-Villeda at sanchezvilledah@missouri.edu
