# Supplementary material for: DNAAlignEditor: DNA alignment editor tool
Source: BMC Bioinformatics. 2008 Mar 19;9:154. doi: 10.1186/1471-2105-9-154 (PMC2322986; doi:10.1186/1471-2105-9-154)
Supplement: Additional file 4 — DNA alignment tool installation instructions. Installation steps for DNAAlignEditor tool. [file 1471-2105-9-154-S4.doc]

File 3. Describes how to install the tool. The DNASetUp.zip and Data_Test.zip files that are needed to install the application and examples to test the software, described in the next steps, must be downloaded at: http://maize.agron.missouri.edu/~hsanchez/DNAAlignEditor/DNASetUp.zip and

http://maize.agron.missouri.edu/~hsanchez/DNAAlignEditor/Data_Test.zip

Note: If you are using Windows x64 edition, ensure that the database dnadb is under c:\program files\DNAAlignEditor\

directory, because windows x64 installs in the c:\program files (x86)\ directory.

Source files are included in the DNAAlignEditor_Source.zip file.

installation.txt

February 2008

DNAAlignEditor (DNA Align Editor Tool) Version 2.0

This document contains information that will help you get the most out of DNAAlignEditor Tool.

To read this file on-screen, use the Page Down and Page Up keys on your keyboard. You can[MDM1] Print the file by choosing the Print command from the File menu.

Sending suggestions, content enhancements, and errors

If you have suggestions for features you would like to see in future versions of DNAAlignEditor Tool or

comments about the current version, please send them to Hector Sanchez-Villeda at:

FAX: (573) 884-7850

E-Mail: sanchezvilledah@missouri.edu

E-Mail: hsanchez7@hotmail.com

Mail Address: 210A Curtis Hall Columbia, MO 65211

Comments about errors, comprehensiveness, or validity of information presented are welcome.

Please send these comments by FAX, mail, or e-mail.

Table of Contents

Section Description

1. Download Application & Data

2. Installation Steps

3. TUTORIAL for the DNAAlignEditor Application

4. Problems during setup

5. Minimum hardware requirements

6. Alignments limitations

7. How to use one-line help

8. Data Base Administrator responsibilities

9. Uninstall

1. Download DNAAlignEditor Application (DNASetUp.zip) and Data Examples of DNAAlignEditor (Data_Test.zip) files from the web-site.

2. Installation Steps

a) Create a new folder in your hard drive and copy the DNASetUp.zip and Data_Test.zip files into it.

b) Extract all the files by un-zipping them. It creates several directories.

c) To install DNAAlignEditor Application double click on SetUp.exe file. It is located under Application, Package in the DNASetUp.zip folder.

d) Follow the instructions by clicking the OK button then click on the COMPUTER icon and accept the default values.

Warnings:

If you get the error message -> "A file being copied is older than the file currently on your system. It is recommended to

keep your existing file. Do you want to keep it?". The answer is **YES**. This error happens when you have an older version

of any dll or ocx or another system file so you should keep the version on your computer.`

Sometimes either the oledb32r.dll is not in Windows/System32 or a Windows application is running using this dll. **IGNORE** the

windows errors, then verify if the dll exists in Windows/System32. If it does not exist, copy the dll file from the Support directory

under application and package directory to your Windows/Sustem32 directory and register it using "regsvr32 oledb32r.dll" under "run command"

located on the start menu.

Verify that the following files are installed under C:\Program Files\DNAAlignEditor\

_DEISREG.ISR

clustalw.exe

DelsL1.isu

dnadb.mdb

DNA_AlignEditor_Tool.exe

DNAALIGNEDITOR.hlp (help file)

DNAEditor.ini

Grid32.OCX

msvcrt.dll

muscle.exe

ReadMe.txt

ST6UNST.000

ST6UNST.txt

Treev32

f) To run the program click on the start menu, then all programs and click on DNAAlignEditor under the DNAAlignEditor tab.

g) Once the system is installed, generate a directory under drive C: named ClustalWork (C:\ClustalWork\).

Note:

The DNAAlignEditor program is looking for a directory C:\ClustalWork when the ReClustal button is clicked,

it opens an output file called Clustal_Input. If the directory is not found the program reports the problem

asking to create the directory. Once the file is generated it is filled with the information found in the

grid to be used for either clustalw.exe or muscle.exe, generating the Clustal_Input.pir or Clustal_Input.msl

accordingly. The generated file contains the clustered information, which is loaded by clicking on Load Clustal

button located next to the ReClustal button. You can modify DNAEditor.ini file by changing in the CLUSTAL section,

the path where clustal.exe or the MUSCLE section, the path where the muscle.exe file reside. Most of the time the

path for Clustalw is: ClustalPath=C:\Program Files\SNPAlignEditor\clustalw.exe -align ... and the path for Muscle

is MusclePath=C:\Program Files\SNPAlignEditor\muscle.exe -out ...(the parameters used for clustalw and muscle are

standard parameters, however you can change them according to your needs).

3. TUTORIAL for the DNAAlignEditor Application

a) Run the DNA_AlignEditor_Tool.exe file click on the start menu then all programs, and click on DNAAlignEditor under DNAAlignEditor tab.

b) Un-zip the Data_Test.zip, and import the Import_Populations.txt file. Click on Catalog, Tab Delimited Files and Populations tab.

In the load populations screen, click on Select Input File button and locate the Import_Populations.txt file, then select an Error Log File

to save the log file error, and click on the OK button.

c) Import the import_Primers-Genes.txt file. Click on Catalog, Tab Delimited Files and Primers-Genes tab.

In the load primers-genes screen, click on Select Input File button and locate the import_Primers-Genes.txt file, then select an Error Log File

to save the log file error, and click on the OK button.

d) Import the Import_Fasta_Hector.fas file. Click on Data and Import alignments tab. In the Import Alignments form click

on Fasta radio button, then click on Gene Name Find button and select the gene by double click on the preferred gene, then

click on Amplicon Name Find button and select the amplicon by double click on the preferred amplicon, click on Validate button, then click on

Select input file button and locate the Import_Fasta_Hector.fas file, then click on Error Log File button and click on OK button.

Note: There are other alignments files that can be imported using the same procedure, you do not need to import populations or primer-genes because

they were already imported in the steps b) and c). Generate your own genes-primers and populations according to your needs in

order to import your fasta files, ace files or standard format files.

e) Click on Data and Edit Alignment Tool to view the alignment loaded and work with the Editor Tool.

4. Problems during setup

Because DNAAlignEditor Tool installs system files that may be shared with other applications, it is best to

shut down other applications before installing. If Setup is interrupted by a loss of power, a power surge,

or some other unexpected incidents, then restart Windows before you run Setup again. If you get the error message ->

c:\windows\system\Mfc42.dll "The destination file in in-use. Please ensure that all other applications are closed,

just ignore the message, then another message will be displayed "If you ignore a copy error, the file will not

be copied. The application may not function properly as a result. Do you want to ignore the error ?. Answer

is **YES**. This happens sometimes because Windows is using the dlls to run itself.

If you get the error message -> "An error occurred while registering the file EXPSRV.DLL", you can ignore it,

but you might need to register using the REGSRV32.exe program. This error happens when the setup program is

trying to register a DLL or OCX into the registry. Sometimes either the odbc32r.dll is not in Windows/System32

or a Windows application is running using this dll. Ignore the windows errors, then verify if the dll exists in

Windows/System32. If it does not exist, copy the dll and register it using "regsvr32 oledb32r.dll" under "run

command" located on the start menu.

5. Minimum hardware requirements

Before installing the system, make sure your computer system meets the following requirements.

Then read the section 3 of this file "Running set up"

Component Client Requirement

Computer Pentium(R) 4 or higher processor

RAM Memory 1 GB; 2 GB is recommended

Disk space 100 MB; 10 GB is recommended

Windows Ms-WINDOWS XP or later

Database AccessDB (It installs the Database, but it does not require MSAccess)

ODBC driver Embedded in the installation

Devices CD ROM or USB driver or 3 1/2" driver or Hard disk

6. Alignments limitations

We programmed the DNAAlignEditor tool with a dynamic grid that can handle as much ram memory as you have in your computer, as far as we know there is no specific limit to the number of sequences and bases you work with. We have successfully tested the application with 84 sequences and 7,086 bases using a DELL Pentium® 4 at 3.4 GHz with 3.5 GB of RAM. Using larger numbers of sequences of greater length will affect performance, depending on the specific processor.

7. How to use on-line help

Once the DNAAlignEditor Tool system is installed, online help is a convenient quick way to look up information

about the task you are performing. Help is available whenever you see a Help command button or

by pressing F1 key.

8. Data Base Administrator responsibilities

The D.B.A. must do the following activities

a. Read help files

b. The D.B.A. must control the users that are allowed to enter the system as well as the

the permissions for each.

c. Install the application on any computer on the network and verify that they are working

properly.

d. Back ups of the information should be made after a certain amount of time has been spent in changing one or

more files.

Database Backup for dnanp.

9. Uninstall package.

To uninstall the DNAAlignEditor tool you need to click on the start button, settings and control panel, then click

on the Add/Remove program icon and look for the DNAAlignEditor, finally click on Change/Remove button

to uninstall it.

Generals:

The Speed of the system for data entry, and generation of reports depends on computer used,

characteristics and hard disk available and networking processes.
